# Correlation of serum metal ion levels with pathological changes of ARMD in failed metal-on-metal-hip-resurfacing arthroplasties

**DOI:** 10.1007/s00402-017-2723-x

**Published:** 2017-06-28

**Authors:** George Grammatopoulos, Mitsuru Munemoto, Athanasios Pollalis, Nicholas A. Athanasou

**Affiliations:** 1 Nuffield Orthopaedic Centre, Windmill Road, Headington, Oxford, OX3 7LD UK; 2 Nuffield Department Orthopaedics Rheumatology and Musculoskeletal Sciences (NDORMS), University of Oxford, Windmill Road, Oxford, UK; 3 Department of Orthopaedic Surgery, Nara Medical University, Kashihara-City, Nara 634-8522 Japan

**Keywords:** Metal-on-metal, ALVAL, ARMD, Pseudotumour, Hip resurfacing, Revision

## Abstract

**Background:**

Metal-on-metal-hip-resurfacing arthroplasties (MoMHRAs) have been associated with an increased failure rates due to an adverse-response-to-metal-debris (ARMD) associated with a spectrum of pathological features. Serum levels of cobalt (Co) and chromium (Cr) are used to assess MoMHRAs, with regard to ARMD, but it is not certain whether ion levels correlate with pathological changes in periprosthetic tissues.

**Methods:**

Serum Co and Cr levels were correlated with histological findings in 38 revised MoMHRAs (29 pseudotumour cases and 9 non-pseudotumour cases revised for pain). The extent of necrosis and macrophage infiltrate as well as the aseptic lymphocyte-dominated vasculitis-associated lesion (ALVAL) response was assessed semi-quantitatively; the prosthesis linear wear rate (PLWR) was also determined in ten cases.

**Results:**

Cr levels were elevated in 82% and Co levels elevated in 53% of cases; the PLWR correlated with Cr level (rho = 0.8, *p* = 0.006). Tissue necrosis and macrophage infiltration were noted in all, most of which also exhibited significant ALVAL. Although a discrete correlation was not seen between Co and/or Cr ion levels and the extent of necrosis, degree of macrophage infiltration, or ALVAL score, it was noted that cases with acceptable metal ions levels had high ALVAL score.

**Conclusion:**

Histological features of both innate and adaptive immune response to metal wear are seen in periprosthetic tissues in cases with both elevated and non-elevated metal ion levels. MoMHRA failures with acceptable ion levels exhibited a pronounced ALVAL response. Although metal ion levels are elevated in most cases of MoMHRA failure due to ARMD, the finding of a normal metal ion level does not exclude this diagnosis.

## Introduction

Metal-on-metal (MoM) total hip and MoM hip-resurfacing arthroplasties (MoMHRAs) have generally been used in younger patients because of theoretical advantages, including the lower wear profile of these implants [1]. A number of these MoM implants have been associated with a relatively high mid-term failure rate due to an adverse reaction to metal debris (ARMD) [2]. MoM hip arthroplasties have a unique characteristic amongst bearing couples in that it is possible to estimate implant wear in vivo by measuring serum metal ion levels [3]. Serum chromium (Cr) and cobalt (Co) levels are higher in patients with MoM hip implants compared with other implant types [3–5], and elevated metal ion levels have been associated with increased wear [6–8]. However, whether elevated metal ion levels correlate with an increase in implant failure due to ARMD remains controversial [9–13].

ARMD is due to the tissue response to MoM implant-derived metal wear debris in periprosthetic tissues [14]. This includes a non-specific innate foreign body response, largely mediated by macrophages, and a specific adaptive cell-mediated reaction characterized by the presence of a perivascular lymphoid infiltrate [15, 16]. The latter has been termed aseptic lymphocyte-dominated vascular-associated lesion (ALVAL) and is thought to be due to a cell-mediated Type IV delayed hypersensitivity response to the products of metal wear modified by interaction with cell and tissue proteins [15, 16]. It is uncertain whether high serum metal ion levels are seen in all cases of MoMHRA failure and whether specific pathological changes of ARMD are seen at both normal and high metal ion levels.

Serum metal ion levels are generally considered to provide a guide to implant status with regard to ARMD. In this study, we have analysed Co and Cr levels in patients with failed MoMHRAs and correlated these measurements with pathological findings in periprosthetic tissues. Our aim has been not only to determine whether all cases of ARMD have high serum metal ion levels and exhibit increased prosthesis wear but also to examine whether metal ion levels correlate with specific histological features indicative of the innate and adaptive immune response seen in ARMD.

## Materials and methods

We analysed serum Co and Cr levels as well as morphological changes in periprosthetic soft tissues of 38 hips (35 patients). Inclusion criteria for revised MoMHRAs to be entered into this IRB approved study included:

1. Patient consent for use of tissue and availability of serum metal ion levels for Co and Cr.
2. Availability of tissue for histological examination.
3. Clinical data and radiological findings [X-ray, ultrasound scan (US), and magnetic resonance imaging (MRI)] for revision mode classification.

36 (95%) hips had the index MoMHRA procedure at our centre. MoMHRAs used included the Birmingham hip resurfacing (*n* = 26) (Smith and Nephew, Leamington Spa, UK) and the conserve hip resurfacing (*n* = 12) (MicroPort, Chester, UK). Both designs have acceptable subtended angles and metallurgy. The majority of revisions were in females (*n* = 21, 68%, 16 patients). All cases underwent arthroplasty for osteoarthritis. The mean age at operation was 54.2 years (range 33.4–69.8) and the mean implant survival was 6.5 years (range 1.5–11.4). The majority of failures were in patients with a unilateral MoMHRA (*n* = 25); 7 failures were in patients that had bilateral MoMHRAs and one side failed; 6 failures were in 3 patients with bilateral MoMHRAs that required bilateral revisions and fulfilled all criteria for inclusion of both sides in this study. Detailed cohort demographics are included in Table 1. The commonest cause of revision (*n* = 29) was the presence of a symptomatic pseudotumour. The remaining cases were revised for pain and had no radiological or intra-operative features of pseudotumour, infection, or other recognized mode of MoMHRA failure (see Table 1).

**Table 1:** Study group demographics

	Cohort	Group		*p* value
		PT	Non-PT	
Age	54 (9) 33–70	54 (9) 33–68	55 (10) 44–70	0.9
Females	26 (68%)	20 (69%)	6 (67%)	0.9
Implant survival/years	7 (3) 2–11	6 (3) 2–11	7 (2) 5–10	0.9
Interval implantation to ion level test/years	5.5 (3) 1–11	5 (3) 1–11	6 (2) 4–10	0.5
Interval ion level test to revision/months	10 (1) 0–37	11 (10) 0–37	8 (8) 0–24	0.4
Median Cr level	9 (16) 1–70	9 (14) 2–60	7 (20) 1–70	0.8
Median Co level	4 (17) 1–67	6 (17) 1–65	2 (21) 1–67	0.4

Data provided are mean (SD) and range

*PT* pseudotumour, *Cr* chromium, *Co* cobalt

### Histological analysis of periprosthetic soft tissues

The specimens submitted for histological analysis included capsule/synovium, femoral and acetabular pseudomembrane, and, where relevant, pseudotumour. The mean number of specimens submitted from each case was six (range 2–13). None of the specimens had histological or microbiological evidence of infection. The senior author who was blinded to the cause of failure examined histology and the results of wear analysis and metal ion levels.

Tissue necrosis and the extent of the inflammatory cell infiltrate in MoMHRA periprosthetic tissues was assessed semi-quantitatively as previously described [16, 17]. The number of macrophages was scored as 0 (absent) 1+ (few), 2+ (many), and 3+ (abundant). Necrosis was scored as 0 (absent), 1+ (scattered small necrotic areas), 2+ (frequent small or large necrotic areas: up to 25% tissue involvement), and 3+ (extensive necrosis: more than 25% tissue involvement). The ALVAL response was graded (1–3) using the Oxford scoring system [17]. Repeatability testing demonstrated highly significant inter (*k* + 0.86, *p* < 0.001) and intra (*k* = 0.74, *p* < 0.001) observer (intra-class) correlation coefficients (*k*).

### Analysis of blood metal ion concentration

The mean interval between MoMHRA procedure and metal ion measurement was 5.4 years (range 1–11), and the mean interval between metal ion measurement and revision surgery was 11 months (range 0–37).

Whole blood sampling to measure Co and Cr ion levels was carried out as previously described by Kwon et al. [18]. As reported by van Der Straeten et al. [19] in their study on MoMHRAs, Cr and Co ion levels were considered high if above 4.6 and 4.0 ppb, respectively, for unilateral hips, or more than 7.0 and 5.0 ppb, respectively, for bilaterally resurfaced hips. All materials used to collect and store samples were chosen for lack of metals investigated in the study. For the purpose of this study, if either the Co and/or ion level was increased the patient was considered to have elevated metal ion levels. If neither the Co or Cr level was elevated, the patient was considered to have metal ion levels that were within the normal range (WNR).

### Wear measurement analysis

In 10 patients, wear measurements of the explanted components were available. Wear analysis was performed using a validated technique with a non-contact, optical coordinate measuring system (Redlux) in a blinded fashion at the Smith and Nephew implant development centre (IDC) (Leamington Spa, United Kingdom) [20].

Measurements obtained included linear wear (µm) of each femoral and acetabular component. This allowed for estimation of total (femoral + acetabular) linear wear. Knowing the survival of each MoMHRA, we were able to calculate the total linear wear rate (TLWR) as: total linear wear rate (µm/year) = total linear wear (µm)/implant survival (years).

### Analysis

The measured ion levels were tested for correlation with the wear measurements obtained.

Revision indications were grouped into those revised for pseudotumour (pseudotumour group) and those revised for unexplained pain (non-pseudotumour group). The serum metal ions and the histological findings for the two groups were assessed and the association between histological findings and metal ion levels was tested.

A power calculation on the sample required for sufficient power was established on previous data on histological findings and wear measurements [17]. It has shown that 18% of revised resurfaced hips with high ALVAL have little wear, whilst 68% of the remaining cases have high ALVAL score and high prosthesis wear.

The Mann–Whitney *U* and Kruskal–Wallis, non-parametric tests were used to calculate the level of statistical significance in the non-normally distributed linear wear amounts and serum metal ion levels with regard to different histological findings. Cross-tabulated data were compared using the Chi-square (*χ* ^2^) test. Spearman’s (rho) correlation coefficient was used to assess correlation between metal ion levels, histological features, ALVAL score, and amount of wear detected and TLWR calculated. Statistical analysis was performed with SPSS statistical programme version 21 (IBM, Illinois, US).

## Results

A post hoc calculation illustrates that to detect a difference in incidence of high ALVAL (>2) according to whether Co level was elevated or not (alpha value 0.05 and power of 80%), 12 patients would have been required.

### Metal ion levels and prosthesis linear wear measurements

**Fig. 1:**
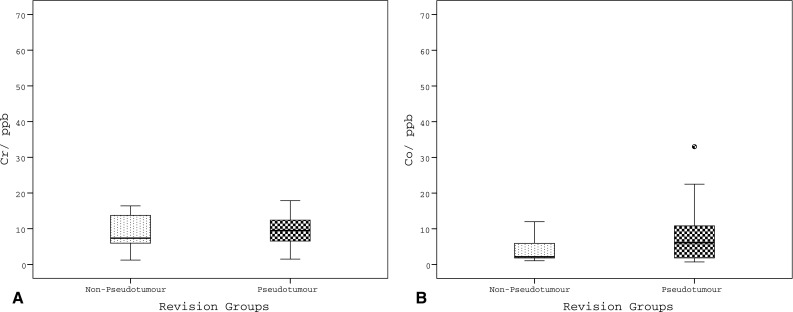
*Box* and *whisker plots* illustrating ion levels measurements of chromium (**a**) and cobalt (**b**) for the different groups as per reason for revision. *Cr* chromium, *Co* cobalt, *ppb* parts per billion

The median level of Cr was 9.1 ppb (1.2–69.5, SD 15.6) and the median level of Co was 4.3 ppb (0.7–67.1, SD 17.6). High levels of Cr were seen in 31 cases (82%). High levels of Co were seen in 20 cases (53%), all of which had a high Cr level. No significant difference in ion levels was detected between the pseudotumour and non-pseudotumour groups (Cr *p* = 0.8; Co *p* = 0.4) (Fig. 1). Most pseudotumour cases were associated with elevated ion levels (23/29, 79%). Similarly, 8 of the 9 non-pseudotumour cases revised for pain were associated with high ion levels.

**Fig. 2:**
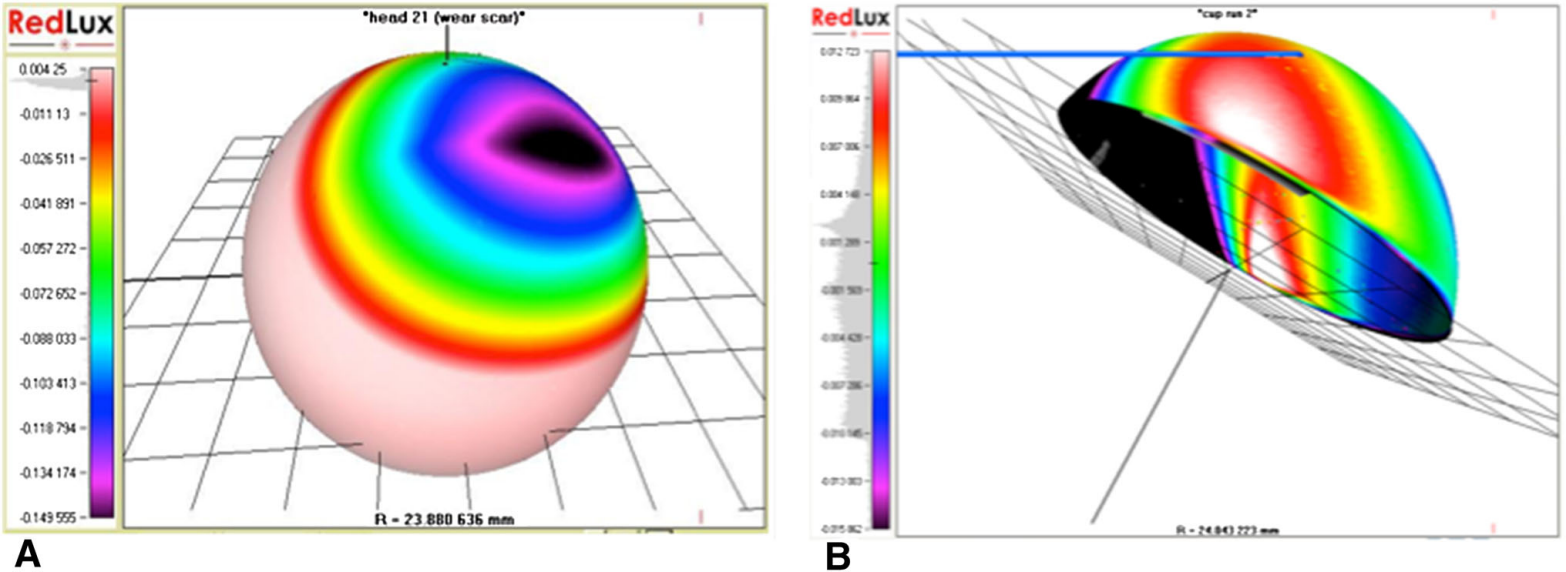
Image of wear analysis illustrating degree of wear (*black* most worn) detected in the surfaces of the **a** acetabular and **b** femoral components

The mean femoral component linear wear was 28 μm (range 9.5–66.9) and the mean acetabular component linear wear was 143 μm (range 0–949). The TLWR was 32.5 µm/year (range 3–201) (Fig. 2a, b). The serum Cr level strongly correlated with TLWR (rho = 0.8, *p* = 0.006), whereas the Co level did not (rho = 0.4, *p* = 0.3).

### Histological findings in periprosthetic MoMHRA tissues

**Fig. 3:**
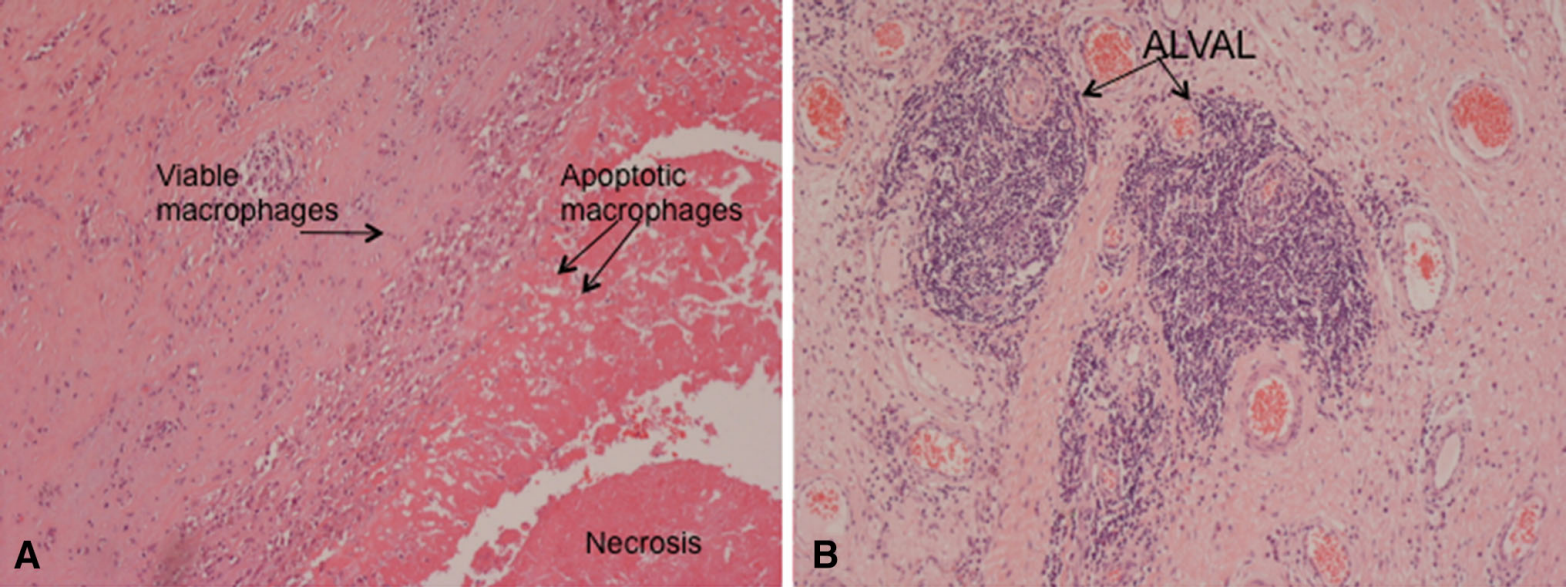
Histology of periprosthetic tissue membrane around failed MoMHRA showing: **a** macrophage infiltrate beneath the surface of the tissue which is covered by necrotic cell and tissue debris containing apoptotic macrophages, **b** Oxford Grade 3 ALVAL response with large perivascular lymphoid aggregates

Necrosis was seen in all cases, the majority of patients showing 2 + (*n* = 7) or 3 + (*n* = 12) necrosis. Necrotic areas were most prominent on the surface of the sampled periprosthetic tissues and often contained numerous apoptotic and necrotic macrophages (Fig. 3a). There were also extensive necrosis and degenerative change in underlying connective tissue of the pseudocapsule. A moderate [2 + (*n* = 3)] or heavy [3 + (*n* = 35)] macrophage infiltrate was noted in all cases. The Oxford-ALVAL score was high (≥2) in most cases (*n* = 32, 84%) (Fig. 3b).

The extent of necrosis, degree of macrophage infiltration, and lymphoid response, as assessed by the Oxford-ALVAL score, were not significantly different between the pseudotumour and the non-pseudotumour cases (*p* = 0.3–0.9) (Table 2). No difference in histological features was seen between unilateral and bilateral MoMHRAs revised for pseudotumour (*p* = 0.2–0.8) (see Table 2).

**Table 2:** Histological features in pseudotumour (PT) and non-PT groups

Histology		Group		*p* value
Feature	Grade	PT	Non-PT	
Necrosis	1	6	3	0.3
	2	12	5	
	3	11	1	
Macrophage infiltration	1	0	0	0.7
	2	2	1	
	3	27	8	
ALVAL score	1	5	1	0.9
	2	7	2	
	3	17	6	

### Correlation of pathological changes with metal ion levels

**Fig. 4:**
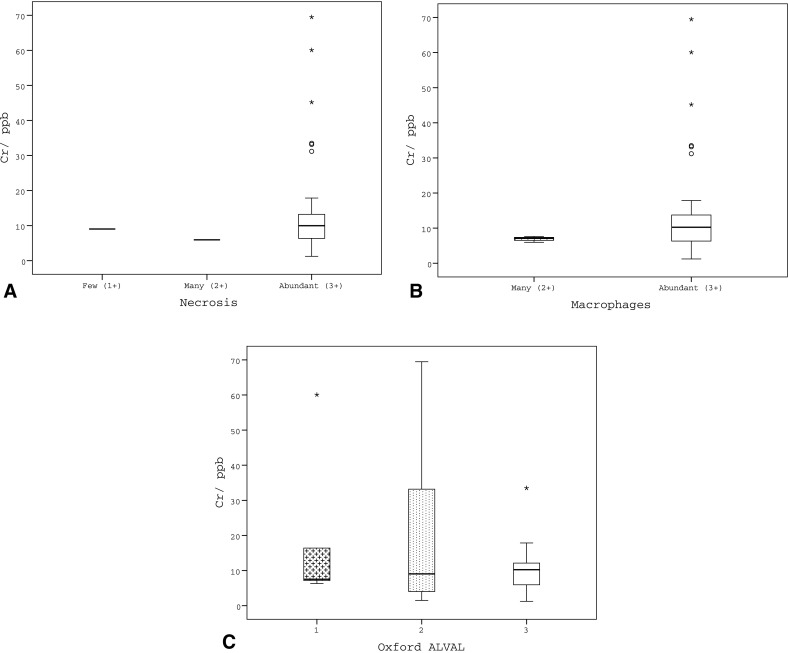
*Box* and *whisker plots* illustrating metal ion levels for the different histological grading groups for necrosis (**a**), macrophage infiltration (**b**), and Oxford-ALVAL response (**c**). *Cr* chromium; *ppb* parts per billion

**Fig. 5:**
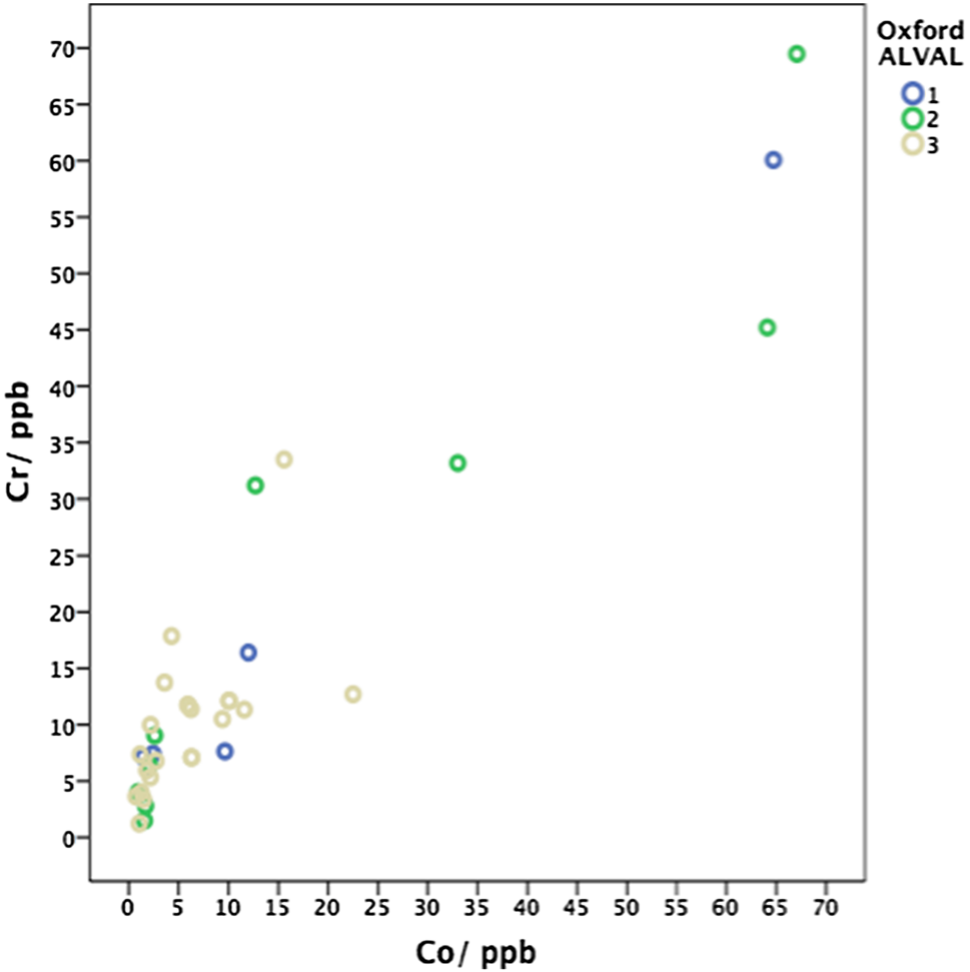
*Scatter plot* of chromium versus cobalt colour-coded for Oxford-ALVAL grade. *Cr* chromium, *Co* cobalt, *ppb* parts per billion

As necrosis and a moderate or heavy macrophage infiltrate was seen in most cases, no discrete correlation was noted between these histological features and Co or Cr levels in the pseudotumour and non-pseudotumour groups (Table 3; Fig. 4). Similarly, as a relatively high Oxford-ALVAL score was seen in most cases of MoMHRA failure, no direct correlation could be made with measured Co or Cr levels, although it was noted that all patients in the pseudotumour and non-pseudotumour groups whose pre-revision metal ion levels were within the normal range had a relatively high Oxford-ALVAL score (2 or 3) (Tables 3, 4; Figs. 4, 5).

**Table 3:** Correlation of histological features with metal ion level groups that were either within the normal range (WNR) or elevated (29)

Histology		Cr			Co		
Feature	Grade	WNR	Elevated	*p* value	WNR	Elevated	*p* value
Necrosis	1	1	8	0.3	4	5	0.8
	2	5	12		9	8	
	3	1	11		5	7	
Macrophage infiltration	1	0	0	0.4	0	0	0.6
	2	0	3		1	2	
	3	7	28		17	18	
ALVAL score	1	0	6	0.3	3	3	0.8
	2	3	6		5	4	
	3	4	19		10	14	

Threshold values; *Cr* 4.6 ppb (unilateral), 7.0 ppb (bilateral) and *Co* 4.0 ppb (unilateral), 5.0 ppb (bilateral)

**Table 4:** Correlation of ALVAL scores with the presence/absence of a pseudotumour (PT) and metal ion (Cr and/or Co) levels that were within the normal range (WNR) or elevated (29)

Revision group	Ion levels	ALVAL score		
		1	2	3
Non-PT	WNR	0	0	1
	Elevated	1	2	5
PT	WNR	0	3	3
	Elevated	5	4	14

Threshold values; *Cr* 4.6 ppb (unilateral), 7.0 ppb (bilateral) and *Co* 4.0 ppb (unilateral), 5.0 ppb (bilateral)

## Discussion

Controversy exists as to whether elevated serum metal ion levels correlate with the degree of implant wear and pathological changes of ARMD [8–11, 13, 20–22]. In this study of MoMHRAs only, we noted that serum Cr and Co levels were elevated in most cases and that the wear rate correlated with the serum level of Cr but not Co. No direct correlation was noted between serum levels of Co and/or Cr and specific pathological changes that reflect the innate and adaptive immune response to metal wear components in ARMD. Most patients with a failed MoMHRA had high serum metal ion levels and showed extensive necrosis, a heavy macrophage infiltrate in response to metal wear particles, and a perivascular lymphoid infiltrate in periprosthetic tissues. No discrete correlation could be established between serum metal ion levels and each of these specific histological features, but it was noted that cases with normal metal ion levels had a high ALVAL score.

Most previous studies on the significance of metal ion levels with regard to ARMD and MoM hip implant failure have correlated Cr and Co levels with implant wear and clinical and/or radiological findings. DeSmet et al. [23] showed a highly significant association in 15 hips between serum Cr and Co ion concentrations and the total linear wear of retrieved MoMHRA femoral components. De Pasquale et al. [24] found that there was a correlation between the presence of metal debris in synovial fluid and metal ion levels in the blood. These studies suggested that measurement of Co and Cr in serum and whole blood provides a guide as to the level of particle release into the joint. Langton et al. [25, 26] also noted a correlation between metal ion levels and volumetric wear in MoMHRAs and concluded that elevated metal ion concentrations are associated with ARMD and consequent early failure. High metal ion levels have also been associated with metal staining of tissues (metallosis), increased wear, and the development of periprosthetic osteolysis [12, 27]. In this study, we examined the correlation of metal ion levels with the linear wear rate rather than total linear wear as in the above studies; this is because measuring the rate of production reduces the effect of run-in wear. Our findings show that serum Cr and not Co correlated with the wear rate. The reason why Cr but not Co showed this correlation is not certain, but it could reflect the fact that Co is more soluble than Cr and enters the circulation more rapidly via diffusion; Co is cleared more quickly than Cr which can accumulate in periprosthetic soft tissues as it binds more avidly to proteins than Co [24, 28–30]. Other factors influencing Co and Cr levels include activity levels, protein synthesis, renal function, positioning of the implant, and implant type. Certain MoMHRAs, such as the ASR resurfacing, are more prone to increased wear, especially at the bearing surfaces, and this has been associated with an increase in metal ion levels [25, 26]. Maezawa et al. also noted an increase in serum and urine Cr but not Co levels in patients with MoM hip arthroplasties [31].

There have been relatively few studies, which have attempted to correlate pathological changes in MoM periprosthetic tissues with metal ion levels. Lohmann et al. [22] found that metal ion levels did not predict the type of tissue response in failed MoM total hip arthroplasties. They noted that the metal content of periprosthetic tissues showed considerable variation but was generally elevated; they also noted that an increase in periprosthetic tissue metal content was associated with a predominantly lymphocytic response, whereas tissues with a lower metal content had a macrophage-dominated response. This observation seems somewhat counter-intuitive given that it would be expected that a heavy metal wear particle load in periprosthetic tissues would elicit a pronounced innate foreign body macrophage response, as noted by Campbell et al. [32] who examined the histology of MoMHRA ‘‘pseudotumours’’ and found considerable variation in the amount and distribution of metal debris; the number, distribution, and type of inflammatory cells; and the degree of tissue necrosis. Whether ALVAL, which reflects the adaptive immune response, is dependent on particle load has not been established, although co-localisation of metal ions in lymphoid aggregates has been reported [33]. In a previous study, we found that the majority of MoMHRA pseudotumours were associated with increased implant wear and that the periprosthetic tissues of most cases showed extensive necrosis, a heavy foreign body macrophage infiltrate, and an ALVAL response [17]. We also noted that a minority of pseudotumours were associated with low wear and a prominent immune response. This finding appears to be mirrored in the present study where we noted that a number of MoMHRA implant failure patients with metal ion levels within the normal range had a high Oxford-ALVAL score.

Our cohort comprised painful hips following MoMHRA with or without pseudotumours as per clinical, radiological, and intra-operative findings. These two groups showed no significant difference in pathological findings, implant survival time (*p* = 0.9), gender (*p* = 0.9), or age (*p* = 0.9). Although we were unable to find a direct correlation between metal ion levels and specific histological features, most patients with elevated ion levels had histological features of extensive tissue necrosis and a macrophage and ALVAL response. It is important to note that some patients with no radiological evidence of a pseudotumour had elevated metal ion levels and histological features of ARMD consistent with a failing MoMHRA. Thus, the absence of radiological findings consistent with pseudotumour should not be taken as evidence that ARMD is not present without having a pseudotumour around their operated hip.

This study has a number of limitations. First, it is a retrospective study design with the associated bias attending this type of study. Only a small number of patients, who had more than one type of MoMHRA implanted, was studied as metal ion testing is not routinely carried out in our institution (selection bias). Second, relatively few patients had prosthesis wear measurements; this was due to limited resources, assessment of bearing wear not forming part of our routine practice. Third, only one set of metal ion levels measurements was available for most patients (89%) and these were not taken a fixed time prior to when the MoMHRA was revised; the interval between metal ion measurement and revision surgery also varied (mean 11 months; range 0–37). In MoMHRAs, there is initially a post-implantation running in period of increased wear followed by steady state and bedding in periods when there is less wear [30]. All measurements in this study were performed during the steady-state period or at short intervals prior to revision, and most revisions were done at mid-term follow-up when wear-related issues are the most-common cause of failure. It has been reported that metal ion levels in MoMHRAs can fluctuate within the steady-state period, and in some cases increase [34]; accordingly, the ion levels reported in this study may be low compared to what the measurement would have been had it been taken at the time of revision. Finally, our methodology did not permit us to establish whether there was a trend towards increasing ion levels.

National regulatory bodies and orthopaedic societies recommend that metal ion levels should be part of the assessment of implant performance [35, 36]. The European consensus statement [35, 36] states that ‘metal ion levels should be performed with every follow-up. Follow-up of resurfacings should be annually for the first 5 years, then according to local protocols for patients with the conventional THR. If metal ion levels are normal at year 1 and 2 postoperatively, the frequency of further annual follow-up investigations may be changed to local protocols for the conventional THR. In patients with risk factors such as small size (<50 mm femoral component) and female gender, MHRA recommends annual follow-up for the lifetime of the implant.

In conclusion, our findings show that there is both an innate and adaptive immune response to metal wear in periprosthetic tissues from failed MoMHRAs and that metal ion levels are elevated in most but not all cases of ARMD. Thus, the finding of a Co and/or Cr ion level within the normal range does not preclude the diagnosis of MoMHRA implant being due to ARMD. Metal ion levels should not be used in isolation to determine whether or not surgical intervention is required for MoMHRA failure due to ARMD; this decision should be based on clinical findings and analysis of radiology and other laboratory investigations.
